# Design of a zinc finger protein binding a sequence upstream of the A20 gene

**DOI:** 10.1186/1472-6750-8-28

**Published:** 2008-03-19

**Authors:** Yong Wei, Dajun Ying, Chunli Hou, Xiaoping Cui, Chuhong Zhu

**Affiliations:** 1 The Key Laboratory of Biomechanics and Tissue Engineering of Chongqing Municipality, Department of Anatomy, Third Military Medical University, Chongqing, 400038, China; 2 Department of Neurology, Fuzhou General Hospital, Fuzhou, 350025, China

## Abstract

**Background:**

Artificial transcription factors (ATFs) are composed of DNA-binding and functional domains. These domains can be fused together to create proteins that can bind a chosen DNA sequence. To construct a valid ATF, it is necessary to design suitable DNA-binding and functional domains. The Cys_2_-His_2 _zinc finger motif is the ideal structural scaffold on which to construct a sequence-specific protein. A20 is a cytoplasmic zinc finger protein that inhibits nuclear factor kappa-B activity and tumor necrosis factor (TNF)-mediated programmed cell death. A20 has been shown to prevent TNF-induced cytotoxicity in a variety of cell types including fibroblasts, B lymphocytes, WEHI 164 cells, NIH 3T3 cells and endothelial cells.

**Results:**

In order to design a zinc finger protein (ZFP) structural domain that binds specific target sequences in the A20 gene promoter region, the structure and sequence composition of this promoter were analyzed by bioinformatics methods. The target sequences in the A20 promoter were submitted to the on-line ZF Tools server of the Barbas Laboratory, Scripps Research Institute (TSRI), to obtain a specific 18 bp target sequence and also the amino acid sequence of a ZFP that would bind to it. Sequence characterization and structural modeling of the predicted ZFP were performed by bioinformatics methods. The optimized DNA sequence of this artificial ZFP was recombined into the eukaryotic expression vector pIRES2-EGFP to construct pIRES2-EGFP/ZFP-flag recombinants, and the expression and biological activity of the ZFP were analyzed by RT-PCR, western blotting and EMSA, respectively. The ZFP was designed successfully and exhibited biological activity.

**Conclusion:**

It is feasible to design specific zinc finger proteins by bioinformatics methods.

## Background

In nature, gene expression is regulated at the transcriptional level primarily by transcription factors that bind to DNA. Many of these transcription factors consist of two essential yet separable modules: a DNA-binding domain and a functional domain [1-3]. Artificial transcription factors (ATFs) are composed of DNA-binding and functional domains [4-6], which can be fused together to create proteins that bind a chosen DNA sequence and regulate expression of a specific gene *in vivo *[1,2,4,7,8]. Construction of ATFs *in vitro *includes construction of DNA-binding and function domains by various methods. It is very important to design a DNA-binding domain that recognizes a specific DNA sequence. Recently there has been a great deal of progress in the development of modular protein domains that recognize specific DNA triplets. The Cys_2_-His_2 _zinc finger motif is the ideal structural scaffold on which a sequence-specific protein may be constructed [7,9]. DNA structural domains of zinc finger proteins (ZFPs) usually consist of 3 or 6 zinc fingers. Artificial ZFP (AZP) technology allows DNA sequences to be selected directionally and a DNA-binding domain to be designed [10-13].

A20 (Tumor Necrosis Factor Alpha-Induced Protein 3, TNFAIP3) is a cytoplasmic zinc finger protein that inhibits nuclear factor kappa-B (NFκB) activity and tumor necrosis factor (TNF)-mediated programmed cell death [14-16]. A20 has been shown to prevent TNF-induced cytotoxicity in a variety of cell types including fibroblasts, B-lymphocytes, WEHI 164 cells, NIH 3T3 cells and endothelial cells [17]. Over-expression of A20 inhibits IL-1β-induced production of NO by rat islets, which impacts positively on islet graft survival and function [18]. Indeed, A20-deficient cells fail to terminate TNF-induced NF-κB activation [19]. Lee *et al*. generated A20-deficient mice by targeted disruption. A20 -/- mice, born from interbred A20 +/- mice in Mendelian ratios, developed runting as early as 1 week of age [13]. Mice deficient in A20 developed severe inflammation and cachexia, were hypersensitive to both lipopolysaccharide and TNF, and died prematurely.

In the present study, to provide a basis for designing ATFs that can regulate the human A20 gene, a ZFP sequence involving six contiguous Cys_2_His_2_-type zinc fingers was constructed using the classical Cys_2_His_2_-type zinc finger model and an 18 bp DNA sequence (as target sequence) obtained from the A20 gene promoter region.

## Results

### Transcription start site and presumptive promoter sequence of A20 gene

The DNA sequence of the human A20 gene (-1000 bp – +300 bp) was used to analyze the promoter region. Trans-acting factors and related elements that may interact with the aforementioned sequence were analyzed by the online TFSEARCH[20], TESS[21] and Gene2Promoter servers[22]. The results indicated many trans-acting factors binding the relevant elements in the gene sequence, with several HSF binding sites at the distal end and 6 SP1 and 2 NF-κB binding sites in the GC-rich region at the proximal end.

**Figure 1:**
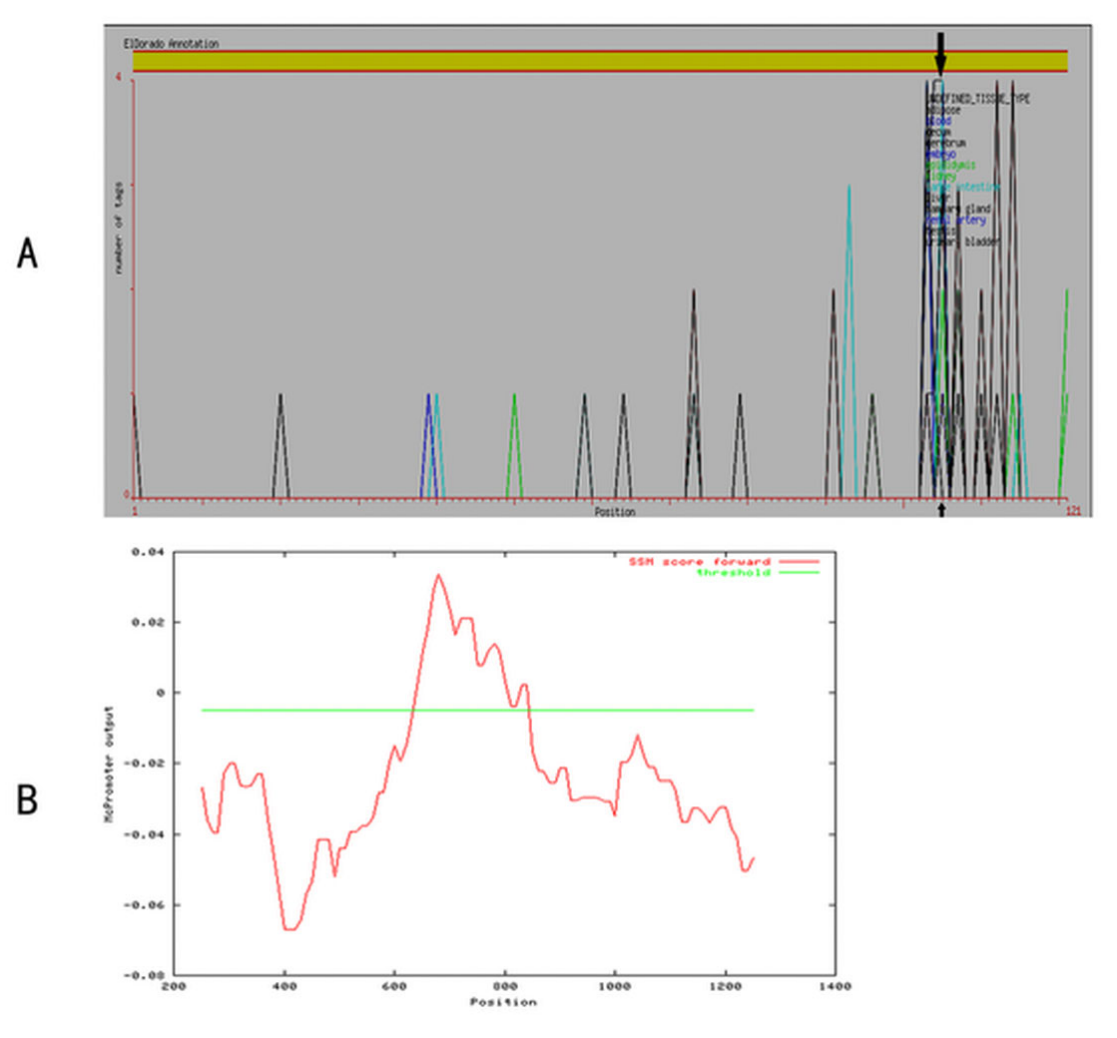
**A: The abscissa represents the region most probably containing the TSS, and the ordinate represents the score for each site.** The higher the score, the more likely the site is to be the TSS. Analysis of the 121 bp sequence showed that the score of a site (located in the genomic NC_000006 138230088) reached a peak value (indicated by arrow). This site was probably the TSS of the A20 gene. **B: **Analysis by McPromoter based on a Markov model suggested that the presumptive promoter of the A20 gene might be located within the genomic NC_000006 138229786 -138230182 and be 396 bp in length. The green straight line represents the threshold value. The part of the score above the threshold suggests a possible presumptive promoter.

The 1300 bp sequence was submitted to Gene2Promoter of Genomatix[22] to locate the A20 gene TSS. The results indicated 12 sequence fragments that may contain TSS sites. The features of these sequence fragments were compared with nucleic acid sequences of different histological origins, and the score of a 121 bp sequence within NC_000006 138229984 -138230104 of the genome proved significantly higher than that of the other sequence fragments. Hence, this 121 bp sequence probably includes the TSS of the A20 gene. Further analysis of the 121 bp sequence showed that the score of the site in genomic NC_000006 138230088 (the site in the frame...agactgcgcagtctg..., see Seq.1) reached a peak (Fig. 1A). Therefore, the TSS of the A20 gene can be determined by bioinformatics methods. The results of prediction were consistent with the conclusions that Krikos drew from an S1 Nuclease Protection Assay [23].

After the TSS of the A20 gene was determined, the established promoter sequence of this gene was submitted to Gene2Promoter of the Genomatix[22] and McPromoter[24] online server (Nuremberg University) to analyze the presumptive promoter sequence further. The results suggested that the presumptive promoter might be located within genomic NC_000006 138229781-138230472 and be 692 bp in length. Moreover, analysis by the McPromoter program based on a Markov model suggested that the presumptive promoter may be a sequence approximately 300 bp in length, which is covered by the sequence predicted by Gene2Promoter (Fig. 1B).

These analyses defined the presumptive promoter sequence and TSS of the A20 gene. In the light of the following study design, a 396 bp sequence fragment within NC_000006 138229786 -138230182 of the A20 gene promoter was selected as the target sequence for study (see Seq.1).

cttccgaaatgcccaggtgactcacgcggggacaccccggggcggggcgagggagtttctccgggcgcctgcagggaccgggcggggcggggcagcggggcggggcagggaaagggggcggggcggggcccgcaggcccggtcgggcggaggccgcgcgcgcccctcgccccctgcgccctctggcggccggctggacgcacttcgcagcccgacccagagagtcacgtgactttggaaagtcccgtggaaatccccgggcctacaacccgcatacaactgaaacggggcaaagcagactgcgcagtctgcagtcttcgtggcgggccaagcgagcttggagcccgcgggggcggagcggtgagagcggccgccaagagagatcacacccccagcc (396 bp)

### Procurement of target sequence of A20 gene promoter region and ZFP design

The presumptive promoter sequence obtained for the A20 gene was submitted to the ZF tools server [25] to "Search DNA Sequence for Contiguous Target Sites". After setting parameters and comparing with the characteristics of ZFPs, we obtained an 18 bp DNA sequence **5'-**cggccggctggacgcact**-3' **(see the sequence in the frame in Seq. 1), which was considered to be the target sequence interacting with the artificial ZFP. This target sequence was then submitted to "Design a Zinc Finger Protein". After setting parameters, we obtained the full-length amino acid sequence of the ZFP (see Seq. 2), which comprised 176 amino acids. In the sequence, the gray bold letters separately refer to the -1, +3 and +6 sites in the α-helix of the zinc finger.

LEPGEKP YKCPECGKSFSTHLDLIRHQRTH

TGEKP YKCPECGKSFSHTGHLLEHQRTH

TGEKP YKCPECGKSFSQRAHLERHQRTH

TGEKP YKCPECGKSFSTSGELVRHQRTH

TGEKP YKCPECGKSFSRNDTLTEHQRTH

TGEKP YKCPECGKSFSRSDKLTEHQRTH

TGKKT S

### Optimization of presumptive nucleotide sequence of ZFP

The presumptive nucleotide sequence (see Seq. 3) of the ZFP was optimized at Graphical Codon Usage Analyser [26].

5-ctggaaccgggtgaaa aaccttacaaatgtccgga atgtggtaaaagcttcagta cccacctggatctgatccgccaccagcgcactcaca ccggcgaaaaaccgtacaagtgcc cagaatgtggcaagagtttcagccacaccggccacc tgctggaacatcagcgtacccataccggtgagaaaccgt acaaatgtcctgaatgcggcaaaagcttt agtcagcgcgcccacctggaacgccaccagcgtacccat accggcgagaaaccgtataagtgtccggaatgcggcaagag ctttagcaccagcggcgaactggtgcgccaccagcgcacccata ccggtgaaaagccatataaatgcccggaatgcggtaaaagtttcagccgcaac gataccctgaccgaacatcagcgtacccacaccggtgagaagccgtataaatgtcca gaatgtggtaagagttttagtcgcagcgataaactgaccgaacaccaacgcacccatacc ggtaaaaagaccagc-3 (528 bp)

### ZFP sequence characterization and structural modeling

**Figure 2:**
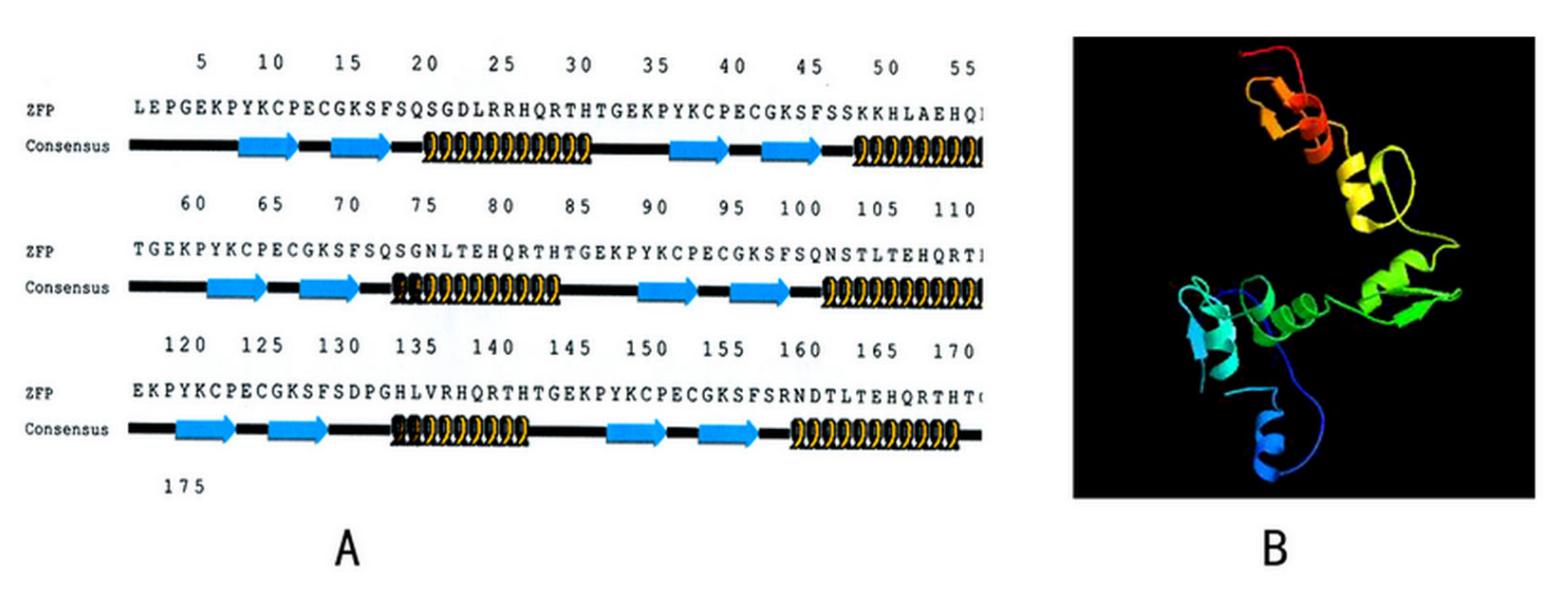
**A: Analysis by PepTool Lite indicated that the 176-amino acid sequence of the ZFP involves 6 regularly-aligned, contiguous Cys_2_His_2_-type zinc fingers, which showed the typical β-β-α secondary structure.B: **Illustration of 6 contiguous β-β-α zinc fingers constituted the 3-D structural modeling of the backbone of the ZFP, carried out on the online Robetta server. This orderly helical structure would be able to bind specially in the DNA major groove.

Analysis by PepTool Lite indicated that the 176-amino acid sequence of the ZFP involves 6 regularly aligned, contiguous Cys_2_His_2_-type zinc fingers, which show the typical β-β-α secondary structure (Fig. 2A). ZFP characterization using ExPASy Proteomics tools – ProtScale suggested that the ZFP has a theoretical isoelectric point of 9.19 and a molecular weight of 19.99 kDa [27,28]. Bulkiness analysis indicated significant decreases in the scores of the dense α-helix of each zinc finger in ZFP, and the scores of the bulky β-fold were the highest [27]. Analysis of polarity and accessible residues also confirmed that the constructed ZFP sequence possessed the structural characteristics and possible biological activity of a typical zinc finger. In the absence of Zn^2+ ^(a stabilizer of the zinc finger structure), the instability index of ZFP was calculated as 42.46, indicating structural instability. The theoretical half-life of the ZFP was predicted to be 5.5 h in eukaryotic cells but only 2 min in *E. coli*. Hence, if the ZFP is to be expressed in prokaryotic cells, protease-deficient bacteria should be used to decrease catabolism of the heterogeneous ZFP; moreover, Zn^2+ ^should be added to the medium to maintain the structural stability of the ZFP.

3-D structural modeling of the ZFP backbone was carried out using the online Robetta server (Washington University)[29,30]. The ZFP file (in PDB format) sent via e-mail was converted by RASWIN software to JPG format. The image showed 6 contiguous zinc fingers, forming an orderly helical structure (Fig. 2B).

### Construction of pIRES2-EGFP/ZFP-flag recombinants

The insert DNA, a 573 bp fragment product amplified by PCR, was subcloned into the plasmid vectors pIRES2-EGFP after double digestion by the restriction endonucleases XhoI and BamHI. The constructed recombinants underwent restriction digest analysis with XhoI and the digestion products were subjected to agarose gel electrophoresis; the result showed a clear electrophoretic band at approximately 5.8 kb, consistent with the theoretical results. Colony PCR analysis and the recombinant sequencing results showed that the construction of pIRES2-EGFP/ZFP-flag was successful.

### Expression of green fluorescent protein by COS-7 cells

**Figure 3:**
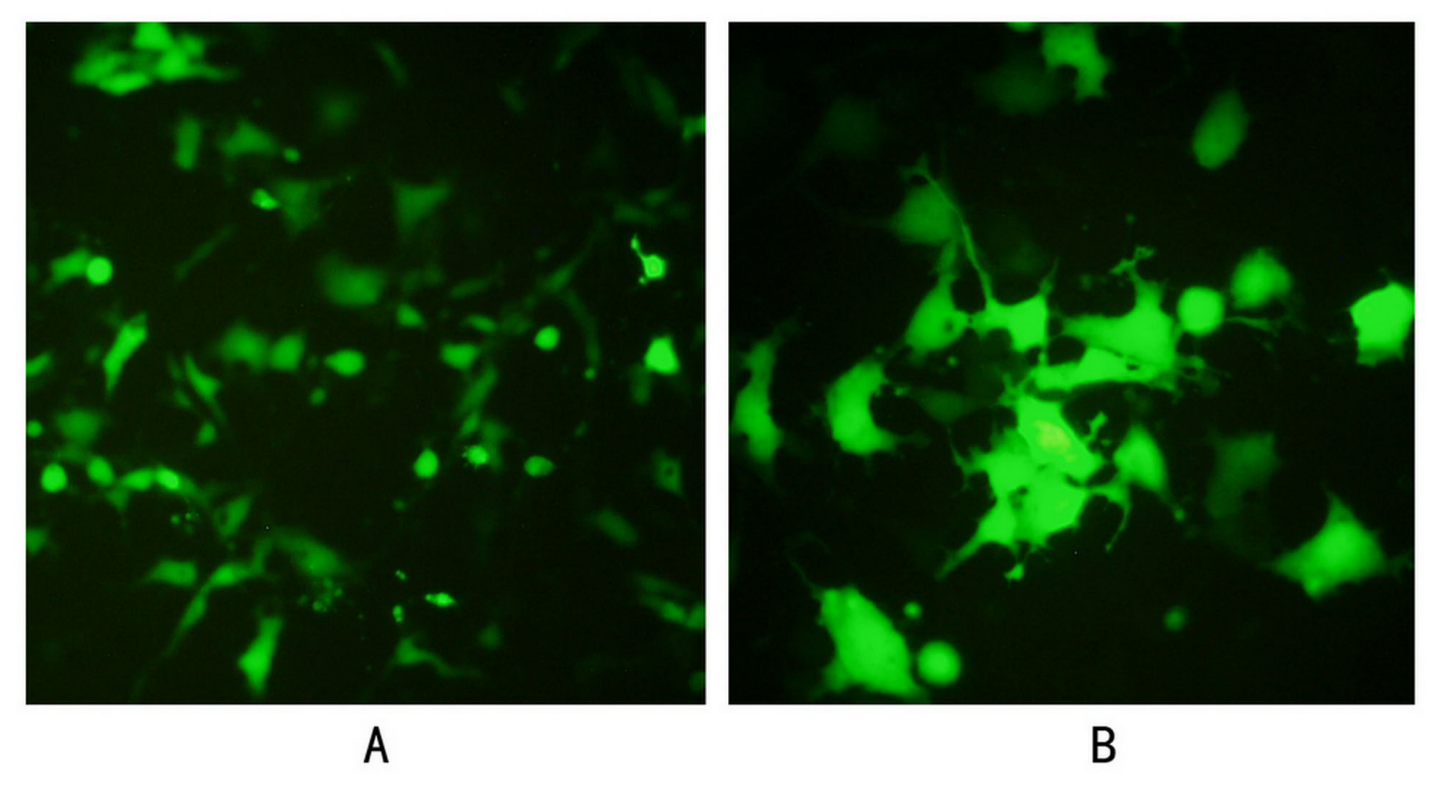
**Expression of green fluorescent protein in COS-7 cells transfected with recombinant plasmids pIRES2-EGFP/ZFP-flag seen through the fluorescence microscope**. **A: **Expression 24 h after transfection (100×). **B: **Expression 36 h after transfection (200×). The result of the pIRES2-EGFP control group was omitted.

In the pIRES2-EGFP/ZFP-flag transfection group, cells emitting green fluorescence were observed under the microscope 12 h after transfection; 24 h after transfection there were significantly more of these cells, up to dozens in each visual field (100×), mostly emitting bright green fluorescence. The fluorescence intensity reached a peak 48 h after transfection, and the cells were found to form masses or pairs, with no significant differences between the nuclear and cytoplasmic fluorescence intensities. In order to determine the transfection efficiency, the cells were counted in 10 random visual fields (100×) 36 h after transfection. Of the 519 cells counted, 227 emitted green fluorescence (43.7%). In the pIRES2-EGFP control group, green fluorescence emitting cells were first observed 8 h after transfection. Moreover, the number of cells emitting green fluorescence and the fluorescence intensity were higher in the pIRES2-EGFP control group than in the pIRES2-EGFP/ZFP-flag transfection group at various times of cell culture. In the control group, no green fluorescence was observed (Fig. 3).

### ZFP mRNA and protein were expressed significantly in COS-7 cells after transfected

**Figure 4:**
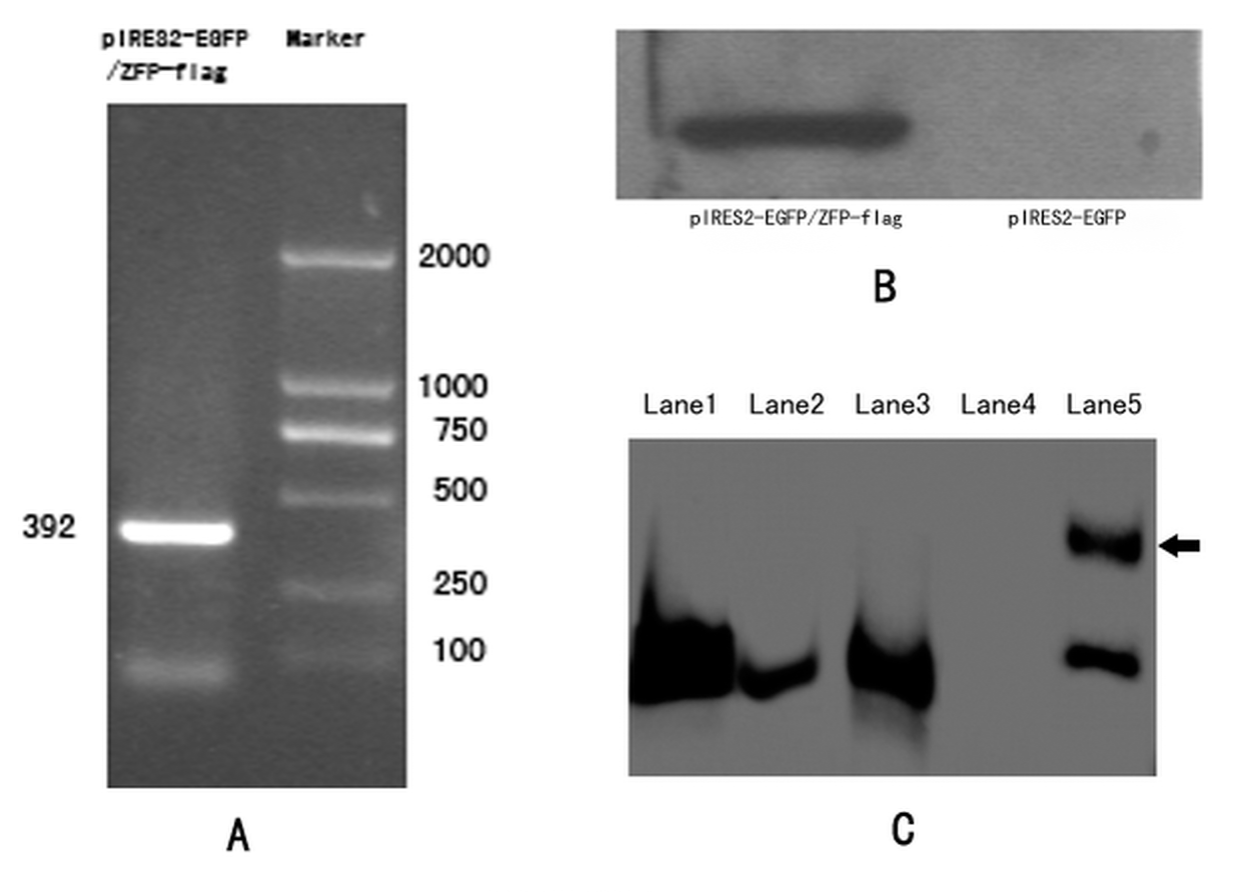
**A: RT-PCR was performed on total RNA isolated from COS7 cells transiently transfected for 24 h with pIRES2-EGFP/ZFP-flag.** Agarose gel electrophoresis indicated an obvious specific amplification band at 392 bp. **B: **Western blotting. The pIRES2-EGFP/ZFP-flag transfection group showed clear protein bands 48 h after transfection, but these did not appear in the pIRES2-EGFP control group. **C: **Electrophoretic mobility shift assay. Lane **1**: sample reaction array. Lane **2**: "cold" competition assay of probes array. Lane **3**: "cold" competition assay of mutated probes array. Lane **4**: negative control reaction array. Lane **5**: Super-Shift reaction array. A lag band of "antigen-antibody-specific probe complex" was observed (indicated by arrow).

Twenty-four hours after plasmid transfection, total RNA was prepared from Cos7 cells. Electrophoresis showed that the total RNA was not degraded. As expected, agarose gel electrophoresis indicated a marked specific band at 392 bp after RT-PCR amplification, consistent with the theoretical results (Fig. 4A). This result was not observed in the pIRES2-EGFP control group. Forty-eight hours after plasmid transfection, Western blotting showed obvious protein bands in the pIRES2-EGFP/ZFP-flag transfection group, but not in the pIRES2-EGFP control group, demonstrating successful expression of the ZFP fusion Flag (Fig. 4B).

### DNA binding activity

EMSA experiments showed corresponding lag bands in the group of protein-specific probe binding reactions (lanes 1 to 3), but no relevant band in the negative control reaction array. In the Super-Shift reaction array, a lag band of the "antigen-antibody-specific probe complex" was observed (Fig. 4C). This experiment demonstrates that the ZFP expressed by COS7 cells can bind to specific probes and possesses biological activity and, at the same time, has a correctly refolded 3-D structure. These findings provide clear evidence for the protein's ability to bind specifically to the target DNA.

## Discussion

With the rapid development of bioinformatics, the volume of biological information in various databases has grown to huge proportions. Most of this information could formerly be obtained only through laboratory research. However, theoretically ideal biological information can now be obtained through efficient use of bioinformatics tools [11]. This has allowed the research tools for modern genetics, biology, biochemistry and pharmacology to expand [31-33].

These developments open new avenues in gene therapy by designing proteins that do not exist naturally but can regulate specific genes by simulating the structure of a naturally-occurring transcription factor. Gene expression is regulated at the transcriptional level primarily (and most economically and effectively) by transcription factors that bind to DNA [3]. To intervene in gene expression, the promoter of the gene of interest must first be studied. A gene promoter contains a series of NA sequence elements adjoining the transcriptional start site (TSS), which directly activate or inhibit transcription. Hence, the position of a promoter can be located through the TSS, once this has been identified. Therefore, precise location of the TSS is the primary requirement for analyzing promoters and transcriptional regulation. The promoter of a gene is frequently located within 100 bp-300 bp upstream of the TSS, and a typical core promoter includes a DNA sequence covering about -40 bp-+50 bp of the TSS [34]. We defined the A20 gene promoter and its TSS theoretically through bioinformatics analysis and showed many potentially important regulatory elements in the A20 promoter region.

An ATF consists of a DNA-binding domain and a functional domain [4-6]. The most crucial step in constructing an effective ATF is to design the DNA-binding domain successfully. Since Miller et al. found that the transcription factor TFIIIA was a Cys_2_His_2_-type zinc finger protein in Xenopus oocytes[35]; hundreds of proteins controlling gene replication and transcription have been found containing zinc finger motif. The Barbas Laboratory developed a series of artificial zinc finger domains by the phage display technique, which recognized different members of the 5'-NNN-3' family of DNA sequences (5'-GNN-3', 5'-CNN-3', 5'-ANN-3', 5'-TNN-3') [9-11,36,37]. They also used them to construct different ATFs. Thus, the Zinc Finger Tools identify individual zinc fingers as a module of the protein's structural domain, and each of the contiguous DNA triplets can specifically recognize an independent zinc finger module [12]. On this basis, as multiple zinc finger modules are integrated into a segment of polypeptide or protein sequence, the resulting sequence can in theory bind a DNA sequence selectively. ZF Tools greatly lower the workload of researchers studying ZFPs. Since the working data of ZF Tools are the results of phage display library screening, the results obtained are theoretically credible; nevertheless, they have to be verified in the laboratory.

Structural modeling of proteins by homologous sequence comparison and alignment is relatively reliable. This method is based on the principle that the 3-D structure of a protein is more conserved than the primary structure[29]. Given this viewpoint, it is essential to acquire the crystal structures of homologous proteins as template. In fact, because of the increasing number of structural determinations of ZFPs, homologous modeling of new proteins containing zinc finger domains may be carried out. We successfully obtained the PDB files of ZFPs from the 3D-JIGSAW, Swiss-Model and Robetta online servers.

## Conclusion

It is feasible to design specific zinc finger proteins by bioinformatics methods. This study provides a basis for artificially designing further complete transcription factors and intervening in the expression of the A20 gene.

## Methods

### DNA synthesis

All the primers and the ZFP gene sequence subcloned in the pUC19 plasmid were synthesized by Shanghai Sangon Biological Engineering Technology & Services Co., Ltd.

### Enzymes and reagents

PCR product purification kits were purchased from Beijing Biodev-Tech, gel recovery kits and plasmid extraction kits from Invitrogen, RT-PCR kits from Hangzhou Boer, and nuclear and cytoplasmic protein extraction kits from Beyotime. RPMI-1640 medium was purchased from Gibco, fetal bovine serum from Hyclone, DOTAP liposomes from Roche, Trizol reagent from Invitrogen, anti-Flag antibody from the Antibody Research Center of Shanghai Institutes for Biological Sciences, and HRP-labeled secondary antibody from Santa Cruz. Taq DNA polymerase and dNTPs were purchased from Promega, restriction endonucleases, T4 DNA ligase and T4 polynucleotide kinase from Takara, prestained standard protein markers from MBI, ZnCl_2_, and nitrocellulose (NC) membrane and X-ray film from Sigma.

### Analysis of A20 gene promoter region

The A20 gene (GeneID:7128) and related sequences were retrieved from the National Center for Biotechnology Information[38]. The A20 gene promoter was analyzed using the sequence processing tools TFSEARCH, TESS, Gene2Promoter and McPromoter.

### Design of presumptive nucleotide sequence of ZFP

We logged on to the Zinc Finger Tools server of the Barbas Laboratory of the Scripps Research Institute, submitted A20 promoter-related sequences, and set up parameters to obtain the specific target site of the A20 gene promoter region and the amino acid sequence of a ZFP. This amino acid sequence was then reverse-translated into a nucleotide sequence, and all codons in that sequence were optimized. The full-length optimized sequence was sent to Shanghai Sangon Company for full-gene synthesis.

### ZFP sequence characterization and structural modeling

The characterization and structural analysis of the ZFP were carried out using PepTool Lite 1.1 and ProtScale. Homologous modeling of the ZFP tertiary structure was conducted using Robetta.

### Construction of pIRES2-EGFP/ZFP-flag eukaryotic expression plasmids

ZFP gene fragments were amplified by PCR, using pUC19-ZFP as a template, and the primers were designed as follows. Forward: 5'-CCGCTCGAGATGGACTACAAGGACGACGATGACAAGCTGGAACCGGGTGA-3'; reverse: 5'-CGGGGATCCGCTGGTCTTTTTACCGGTATGC-3' (framed: restriction sites; underlined: Flag tag-encoding sequence). PCR products and pIRES2-EGFP plasmids were doubly-digested with the restriction endonucleases XhoI and BamHI. According to the design protocol, T_4 _DNA ligase was used to ligate the target fragments, and the ligated products were used to transform competent TOP10 *E. coli*. The recombinants were preliminarily identified by restriction digest analysis and colony PCR (PCR primers, forward: 5'-CCCAGAATGTGGCAAGAGTT-3', reverse: 5'-TGCGTTGGTGTTCCGTCAGT-3'). The bacterial suspensions containing positive recombinants were sent to Shanghai Sangon Company for sequencing. The recombinants identified were named pIRES2-EGFP/ZFP-flag.

### Transfection of COS-7 cells

COS-7 cells were routinely cultured in 24-well plates in RPMI-1640 medium supplemented with 7% fetal bovine serum, 0.1 mM (final concentration) ZnCl_2 _at 37°C and 6%CO_2 _in a humidified atmosphere. At 60% cell confluence, plasmid transfection was carried out according to the instructions on the DOTAP kit. In the experimental group, COS-7 cells were transfected with the recombinant pIRES2-EGFP/ZFP-flag plasmids, and in the positive control group the cells were transfected with void pIRES2-EGFP plasmids. In the blank group, liposomes instead of plasmids were added.

### Analysis of ZFP mRNA expression using RT-PCR

Cos7 cells were transfected for 24 h with the recombinant pIRES2-EGFP/ZFP-flag plasmids. Total RNA was isolated with Trizol Reagent (Invitrogen) according to the manufacturer's instructions. RNA purity was assessed by electrophoresis on formaldehyde-denaturing agarose gels and measuring the OD_260_/OD_280 _absorption ratio. Five micrograms of total RNA was used to synthesize the first strand cDNA under the conditions recommended by the manufacturer. Ten percent of the first strand cDNA was used as template for the PCR. The primers used for RT-PCR amplification were the same as those used for colony PCR. The amplified fragment should be 392 bp in length. PCR amplification products were identified by 1.5% agarose gel electrophoresis.

### Analysis of ZFP protein expression using western blotting assay

Forty-eight hours after plasmid transfection, the cells were collected and lyzed to extract total cellular protein. The processed protein samples were subjected to SDS-polyacrylamide gel electrophoresis and transferred on to a nitrocellulose membrane. The membrane was incubated overnight with anti-Flag monoclonal antibody at 4°C and with horseradish peroxidase (HRP)-labeled goat anti-mouse secondary antibody at room temperature for 2 h. Cells transfected with void pIRES2-EGFP plasmids were used as control.

### Electrophoretic mobility shift assay (EMSA)

On the basis of the target sequence selected from the A20 gene promoter region, a pair of specific probes was designed as follows: ATCCCGGCCGGCTGGACGCACTTACA, and a pair of mutated probes was also designed as follows: ATCCTGGCCGGCTAGACGCACTTACA. The specific probes were labeled with [γ-^32^P]-ATP (3,000Ci/mmol at 10mCi/ml) and purified. Forty-eight hours after plasmid transfection, cell protein was extracted. Electrophoretic mobility shift assays were conducted in accordance with the protocol on the kit.

## Abbreviations

ATF, Artificial transcription factors; TNFAIP3, Tumor Necrosis Factor, alpha-Induced Protein 3; TNF, Tumor necrosis factor; NFκB, Nuclear factor kappa-B; ZF, Zinc finger; ZFP, Zinc finger protein; AZP, artificial zinc finger protein; TSS, Transcription start site; EGFP, Enhanced Green Fluorescent Protein; HRP, Horseradish peroxidase; EMSA, Electrophoretic mobility shift assay.

## Authors' contributions

YW analyzed  the structure and the sequence composition of  the A20 gene, designed the ZFP structural domain by bioinformatic means, and YW performed the construction of pIRES2-EGFP/ZFP-flag recombinant. CHZ conceived the study, and helped in the writing of the manuscript. CLH and XPC performed the COS7  cell culture and transfection, including Rt-PCR, Western blot, with the help of YW. DJY gave advice in the proceeding of  the experiment and edited the manuscript. All authors read and approved the final manuscript.
